# Response and Disease Dynamics in Untreated Metastatic Colorectal Cancer With Bevacizumab-Based Sequential vs. Combination Chemotherapy—Analysis of the Phase 3 XELAVIRI Trial

**DOI:** 10.3389/fonc.2022.751453

**Published:** 2022-02-18

**Authors:** Annika Kurreck, Volker Heinemann, Ludwig Fischer von Weikersthal, Thomas Decker, Florian Kaiser, Jens Uhlig, Michael Schenk, Jens Freiberg-Richter, Bettina Peuser, Claudio Denzlinger, Ullrich Graeven, Kathrin Heinrich, Swantje Held, Arndt Stahler, Annabel Helga Sophie Alig, Ivan Jelas, Jobst C. von Einem, Sebastian Stintzing, Clemens Giessen-Jung, Dominik P. Modest

**Affiliations:** ^1^Charité – Universitätsmedizin Berlin, corporate member of Freie Universität Berlin and Humboldt-Universität zu Berlin, Department of Hematology, Oncology, and Tumor Immunology Charité Virchow Klinikum (CVK), Berlin, Germany; ^2^ Department of Medicine III, University Hospital, Ludwig-Maximilians-Universität (LMU) Munich, München, Germany; ^3^German Cancer Consortium (DKTK), Deutsches Krebsforschungszentrum (DKFZ), Heidelberg, Germany; ^4^Gesundheitszentrum St. Marien, Amberg, Germany; ^5^Oncological Practice, Ravensburg, Germany; ^6^Oncological Practice, Landshut, Germany; ^7^Oncological Practice, Naunhof, Germany; ^8^Department of Hematology and Oncology, Clinic “Barmherzige Brüder Regensburg”, Regensburg, Germany; ^9^Oncological Practice, Dresden, Germany; ^10^Oncological Practice am Diakonissenhaus, Leipzig, Germany; ^11^Department of Internal Medicine III (Oncology, Hematology, Palliative Care) Marienhospital, Stuttgart, Germany; ^12^Department of Hematology, Oncology, and Gastroenterology, Kliniken Maria Hilf GmbH, Mönchengladbach, Germany; ^13^ClinAssess GmbH, Leverkusen, Germany; ^14^Charité – Universitätsmedizin Berlin, Corporate Member of Freie Universität Berlin and Humboldt-Universität zu Berlin, Department of Hematology, Oncology, and Tumor Immunology Campus Charité Mitte (CCM), Berlin, Germany

**Keywords:** metastatic colorectal cancer (CRC), disease dynamics, depth of response, early tumor shrinkage, combination chemotherapy

## Abstract

**Introduction:**

Early tumor shrinkage (ETS), depth of response (DpR), and time to DpR represent exploratory endpoints that may serve as early efficacy parameters and predictors of long-term outcome in metastatic colorectal cancer (mCRC). We analyzed these endpoints in mCRC patients treated with first-line bevacizumab-based sequential (initial fluoropyrimidines) versus combination (initial fluoropyrimidines plus irinotecan) chemotherapy within the phase 3 XELAVIRI trial.

**Methods:**

DpR (change from baseline to smallest tumor diameter), ETS (≥20% reduction in tumor diameter at first reassessment), and time to DpR (study randomization to DpR image) were analyzed. We evaluated progression-free survival and overall survival with ETS as stratification parameter according to treatment arm, molecular subgroup, and sex.

**Results:**

In 370 patients analyzed, a higher rate of ETS (60.9% vs. 43.5%; *p* = 0.001) and significantly greater DpR (-40.0% vs. -24.7%; *p* < 0.001) were observed in the initial combination therapy arm. The improvement was pronounced in *RAS*/*BRAF* wild-type tumors. ETS correlated with improved survival irrespective of treatment arm (PFS: *p* < 0.001; OS: *p* = 0.012) and molecular subgroup (PFS: *p* < 0.001; OS: *p* < 0.001). Male patients in contrast to female patients with ETS had survival benefit (PFS: *p* < 0.001, HR 0.532; OS: *p* < 0.001, HR 0.574 vs. PFS: *p* = 0.107; OS: *p* = 0.965).

**Conclusions:**

Initial irinotecan-based combination therapy with bevacizumab improved ETS and DpR in mCRC patients with a particularly high irinotecan sensitivity of RAS/BRAF wild-type tumors. ETS seems to be a suitable prognostic marker for fluoropyrimidine- and bevacizumab-based combinations in mCRC. This finding was rather driven by male patients, potentially indicating that ETS might be less predictive of long-term outcome in an elderly, female population.

## Introduction

Standard systemic therapy in patients with metastatic colorectal cancer (mCRC) usually consists of oxaliplatin- or irinotecan-based doublet or triplet chemotherapy supplemented by monoclonal antibodies according to molecular subtype and primary tumor location (1–9).

The efficacy of treatment is typically evaluated by survival endpoints, such as overall survival (OS) and progression-free survival (PFS). Unlike survival endpoints, the objective response rate (ORR) represents an early study endpoint, but is barely used in phase 3 trials, which is mostly due to the fact that the correlation of ORR with survival is uncertain. An important limitation of ORR, especially in mCRC, appears to be the categorization of responses according to RECIST ignoring more differentiated assessments, in particular the use of parameters indicating early treatment response, such as early tumor shrinkage (ETS) and depth of response (DpR). These parameters enable an early identification of treatment-sensitive tumors and are known to be associated with long-term survival (3, 10–17). The relevance of parameters indicating early treatment response in mCRC has been evaluated by means of ETS and DpR particularly for epidermal growth factor receptor (EGFR) antibody-based regimens (3, 11, 15–17). The impact of irinotecan or other chemotherapeutic agents on these early study endpoints remains less clear.

The XELAVIRI study (AIO KRK-0110) compared the efficacy of fluoropyrimidine (FP) and bevacizumab (Bev) followed by sequential escalation to irinotecan (Iri), FP, and Bev (arm A) with an upfront combination therapy consisting of FP, Iri, and Bev (arm B) in mCRC patients (18). The study concept allows for the investigation of early irinotecan efficacy. In this regard, the underlying analysis aims to evaluate to which extent irinotecan impacts parameters indicating early treatment response and disease dynamics (DpR, ETS, and time to DpR) within the XELAVIRI trial. To further elucidate the impact of irinotecan in mCRC, we analyzed the patient cohort according to *RAS* and *BRAF* mutational status, and sex with special focus on the predictive and prognostic value of the aforementioned parameters.

## Methods

### Patients

We performed a retrospective analysis of the randomized phase 3 XELAVIRI trial evaluating treatment strategies in patients with untreated metastases of colorectal cancer. The trial comprised a total of 421 patients with 212 patients receiving fluoropyrimidine and bevacizumab followed by sequential escalation to irinotecan, fluoropyrimidine, and bevacizumab (arm A) and 209 patients receiving upfront combination therapy with irinotecan, fluoropyrimidine, and bevacizumab (arm B). Detailed treatment schedules are listed in the **Supplementary Table A.1**.

For information concerning trial design and conduct, Declaration of Helsinki, etc. please refer to ClinicalTrials.gov, NCT01249638 and the primary publication (18). The last update on response and survival endpoints was conducted in July 2020.

A clinical database was established for patients that had evaluable DpR data. Tumor samples were tested for *KRAS, NRAS*, and *BRAF* mutations as described previously (18).

### Disease Assessments

Computed tomography of chest and abdomen was performed within 4 weeks prior to start of study treatment. During active study therapy, computed tomography was conducted every 9 weeks until the end of treatment. During follow-up after study treatment, tumor assessments were scheduled every 3 months until the patient’s death or up to a maximum of 5 years.

### Definition of Depth of Response and Early Tumor Shrinkage

DpR was defined as the relation of smallest tumor diameter to baseline tumor diameter. The development of new lesions was evaluated as an increase of 100% in diameter. ETS was defined as at least 20% reduction in tumor diameter at first reassessment (9 weeks from therapy initiation).

### Time to Depth of Response Assessment

Time to DpR was defined as time from randomization to the date of DpR. The analysis was limited to patients with a DpR ≤0%.

### Definition of Progression-Free Survival and Overall Survival

PFS was defined as time from randomization to first progression of disease or death from any cause (whatever occurred first). Overall survival was defined as time from randomization to death from any cause. Patients without progression or death were censored at the last day of follow-up.

### Association of Early Tumor Shrinkage with Survival Endpoints

PFS and OS were evaluated with ETS as stratification parameter (ETS vs. no-ETS) according to treatment arm, molecular subgroups, and sex. Age, treatment arm, sex, *RAS* mutation, and *BRAF* mutation were used as covariates.

### Statistical Analysis

All statistical analyses were performed using SPSS version 25.0 software (IBM Corporation, Armonk, NY, USA).

For univariate analyses, Fisher’s exact tests or chi-square tests were used to evaluate differences between groups, and corresponding odds ratios with 95% confidence intervals were indicated. DpR was compared with non-parametric test (Mann–Whitney *U*). Survival was expressed as medians by Kaplan–Meier method including 95% confidence intervals and compared by log-rank testing as well as Cox regression. The two-sided significance level was set to 0.05 with a 95% confidence interval.

## Results

### Patient and Tumor Characteristics

Out of 421 patients in the modified intention-to-treat population (mITT), DpR and ETS were available for 370 patients [186/212 (87.7%) of patients in the sequential treatment arm and 184/209 (88.0%) of patients in the initial combination treatment arm]. Information on the molecular subtype (*RAS* and *BRAF* status) was available for 330 of these 370 patients. Within the population evaluable for response, one tumor was characterized as both *RAS* and *BRAF* mutant (*BRAF* MT) and was consecutively analyzed within the *BRAF* MT cohort.

A consort diagram illustrating the study population is shown in **Supplementary Figure A.1**. Baseline patient and tumor characteristics are summarized in **Table 1**.

**Table 1:** Patient and tumor characteristics in patients assessable for early response parameters.

Characteristics	Sequential treatment arm	Initial combination treatment arm
*N*, %	(*N* = 186)	(*N* = 184)
**Sex**		
Male	117 (62.9%)	125 (67.9%)
Female	69 (37.1%)	59 (32.1%)
**ECOG**		
0	112 (60.2%)	112 (60.9%)
1	73 (39.2%)	70 (38.0%)
Unknown	1 (0.5%)	2 (1.1%)
**Age**		
Median years (range)	72 (43-86)	69 (42-88)
**RAS/BRAF status**		
RAS/BRAF wild type	69 (37.1%)	73 (39.7%)
RAS mutant	83 (44.6%)	85 (46.2%)
BRAF mutant	11 (5.9%)	9 (4.9%)
Unknown	23 (12.4%)	17 (9.2%)
**Primary tumor side**		
Left	127 (68.3%)	123 (66.8%)
Right	57 (30.6%)	56 (30.4%)
Unknown	2 (1.1%)	5 (2.7%)
**Onset of metastases**		
Synchronous	133 (71.5%)	130 (70.7%)
Metachronous	49 (26.3%)	50 (27.2%)
Unknown	4 (2.2%)	4 (2.2%)
**No. of metastatic sites**		
≥ 2	114 (61.63%)	109 (59.2%)
**Metastatic spread**		
Liver	138 (74.2%)	142 (77.2%)
Liver-limited	47 (25.3%)	53 (28.8%)
Lung	97 (52.2%)	74 (40.2%)
Lymph nodes	65 (34.9%)	73 (39.7%)
Peritoneum	12 (6.5%)	8 (4.3%)
Others	39 (21.0%)	32 (17.4%)
**Laboratory parameters**		
Leukocytes		
≥8,000/μl	85 (45.7%)	83 (45.1%)
Alkaline phosphatase		
≥300 U/L	25 (13.4%)	22 (12.0%)
**Reported prior treatment**		
Radiotherapy		
Yes	35 (18.8%)	29 (15.8%)
No or unknown	151 (81.2%)	155 (84.2%)
Adjuvant chemotherapy		
Yes	46 (24.7%)	42 (22.8%)
No or unknown	140 (75.3%)	142 (77.2%)

Sequential treatment arm: fluoropyrimidine plus bevacizumab; initial combination treatment arm: fluoropyrimidine, bevacizumab, and irinotecan. ECOG, performance status according to Eastern Cooperative Oncology Group.

### Early Tumor Shrinkage and Depth of Response

In the initial combination arm, patients achieved a significantly greater DpR (-40.0% vs. -24.7%; *p* < 0.001) and a higher rate of median ETS [60.9% vs. 43.5%; OR 2.00 (95% CI: 1.33–3.03); *p* = 0.001] at time of first radiological reassessment as compared to patients in the sequential treatment arm. These differences remained statistically significant in multivariate analysis using *BRAF* MT, *RAS* MT, age, and sex as covariates [DpR: *p* < 0.001; ETS: OR 5.68 (95% CI: 3.57–13.16); *p* = 0.001].

With regard to mutational status, patients with *RAS* wild-type (*RAS* WT) and *BRAF* wild-type (*BRAF* WT) mCRC demonstrated a significantly greater median DpR (-49.6% vs. -29.3%; *p* < 0.001) and a higher frequency of ETS [72.6% vs. 50.7%; OR 2.56 (95% CI: 1.28–5.26); *p* = 0.002] when receiving upfront combination therapy. Treatment arm remained an independent factor for improved DpR (*p* = 0.009) and ETS [OR 5.53 (95% CI: 2.99–34.48); *p* = 0.020] in *RAS/BRAF* WT patients.

In univariate analysis, patients with *RAS* MT mCRC benefitted significantly from initial combination treatment in terms of DpR (-33.3% vs. -19.4%; *p* = 0.01), however, without reaching statistical significance in multivariate analysis (*p* = 0.077). The differences in ETS between both therapy arms did not reach statistical significance in the subgroup of *RAS* MT patients.

There were no significant differences in DpR and ETS of *BRAF* MT patients between the respective treatment arms.

The male population in contrast to female mCRC patients significantly benefitted from the initial combination treatment in terms of median DpR (male: -40.0% vs. -22.2%; *p* < 0.001; female: -34.0% vs. -24.4%; *p* = 0.13) and rate of ETS [male: 64.8% vs. 40.2%; OR 2.78 (95% CI: 1.64–4.55); *p* < 0.001; female: 52.5% vs. 49.3%; *p* = 0.73]. These differences remained statistically significant in multivariate analysis [DpR: *p* < 0.001; ETS: OR 4.24 (95% CI: 2.78–8.93); *p* < 0.001].

**Figure 1:**
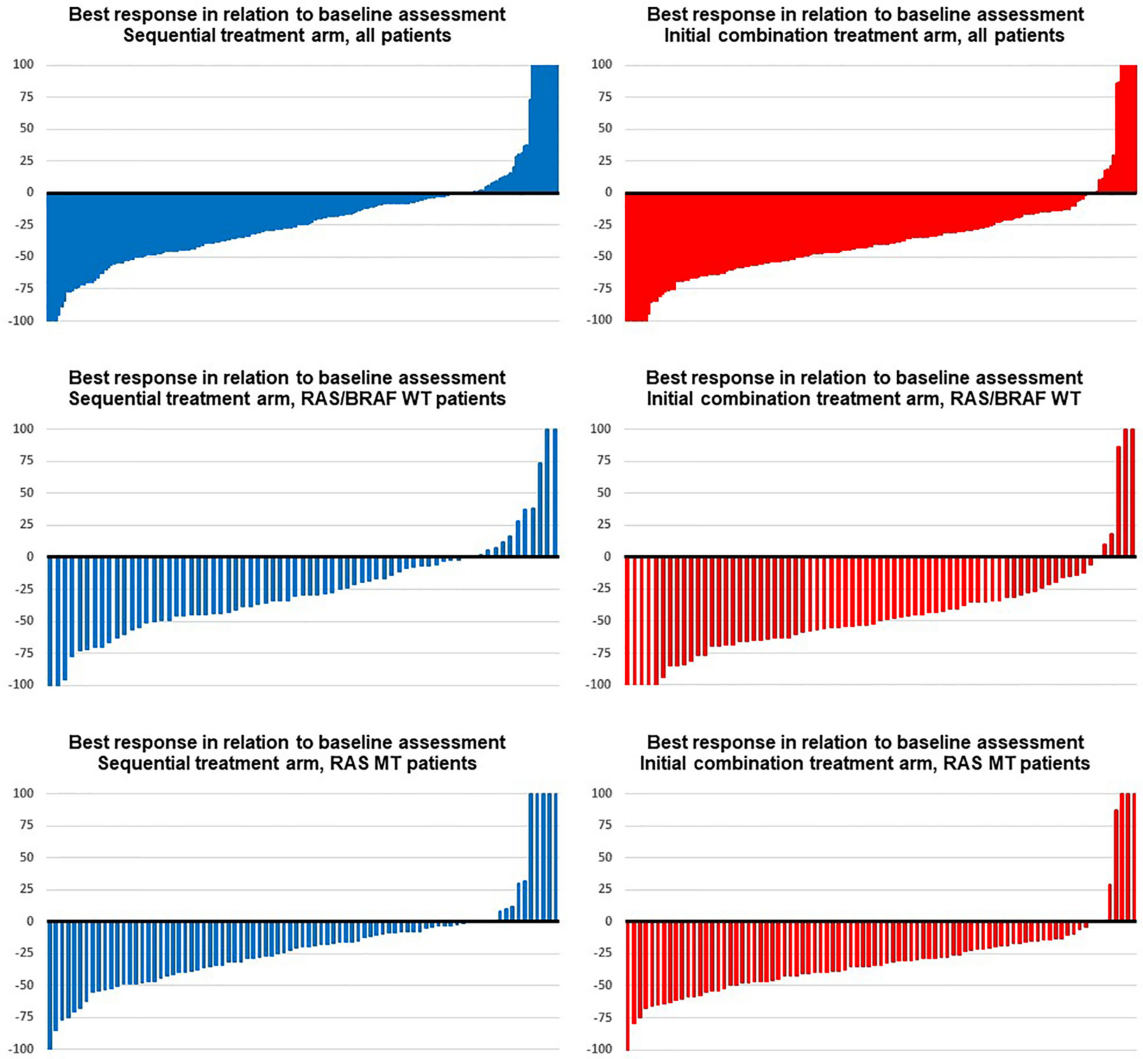
Best response in the trial. Blue images display response assessments of the sequential treatment arm (fluoropyrimidine plus bevacizumab), and red images show response assessments of the initial combination treatment arm (fluoropyrimidine, bevacizumab, and irinotecan) in (from top to bottom) groups: all patients, RAS/BRAF wild type, RAS mutant.

Detailed information concerning DpR and rate of ETS are summarized in **Figure 1**, **Table 2**, and **Supplementary Figure A.2**.

**Table 2:** Parameters of early treatment response in therapy arms according to tumor mutational status and sex.

Population	ETS (≥20% at 9 weeks)			Depth of response	
	ETS in %	OR (95% CI)	*p*-value¹	DpR in % (range)	*p*-value^²^
**Response evaluable population**					
Sequential arm (*N* = 186)	43.5	2.00 (1.33–3.03)	*p* = 0.001	−24.7 (−100–100)	*p* < 0.001
Initial combination arm (*N* = 184)	60.9			−40.0 (−100–100)	
***RAS/BRAF* wild-type group**					
Sequential arm (*N* = 69)	50.7	2.56 (1.28–5.26)	*p* = 0.01	−29.3 (−100–100)	*p* < 0.001
Initial combination arm (*N* = 73)	72.6			−49.6 (−100–100)	
***RAS* mutant group**					
Sequential arm (*N* = 83)	39.8	1.70 (0.93–3.13)	*p* = 0.09	−19.4 (−100–100)	*p* = 0.01
Initial combination arm (*N* = 85)	52.9			−33.3 (−100–100)	
***BRAF* mutant group**					
Sequential arm (*N* = 11)	45.5	0.96 (0.16–5.56)	p=1.0	−10.5 (−88.2–100)	*p* = 0.65
Initial combination arm (*N* = 9)	44.4			−43.0 (−75.2–100)	
**Female patients**					
Sequential arm (*N* = 69)	49.3	1.14 (0.57–2.27)	*p* = 0.73	−24.4 (−100–100)	*p* = 0.13
Initial combination arm (*N* = 59)	52.5			−34.0 (−100–100)	
**Male patients**					
Sequential arm (*N* = 117)	40.2	2.78 (1.64–4.55)	*p* < 0.001	−22.2 (−100–100)	*p* < 0.001
Initial combination arm (*N* = 125)	64.8			−40.0 (−100–100)	

Sequential arm: fluoropyrimidine plus bevacizumab; initial combination arm: fluoropyrimidine, bevacizumab, and irinotecan. DpR, Depth of response expressed as median percentage; ETS, early tumor shrinkage of at least 20% at first reassessment; RAS, rat sarcoma; BRAF, v-raf murine sarcoma viral oncogene homolog B. ¹ Fisher’s exact test; ² Mann–Whitney U test.

### Time to Depth of Response

**Figure 2:**
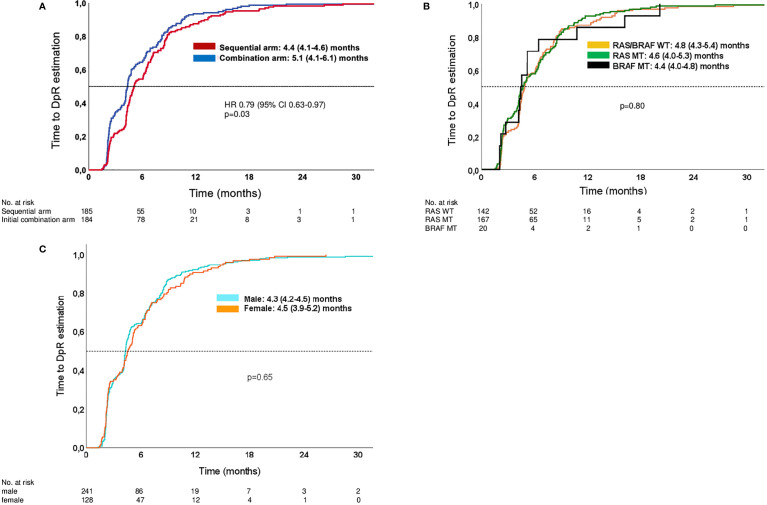
Kaplan–Meier estimates of time to DpR. **(A)** Time to DpR in study arms. **(B)** Time to DpR in molecular subgroups (both arms of study). **(C)** Time to DpR in male and female patients (both arms of study). Analyses are limited to patients with a DpR of at least 0% (no change) or reduction in tumor diameter.

Patients treated within the sequential therapy arm had a significantly shorter time to DpR compared to patients in the upfront combination treatment arm [4.4 months (95% CI: 4.1–4.6 months) vs. 5.1 months (95% CI: 4.1–6.1 months); *p* = 0.03]. Within the different molecular subgroups and genders, the time to DpR was comparable. **Figure 2** contains Kaplan–Meier curves estimating the time to DpR.

### Correlation of Early Tumor Shrinkage with Progression-Free Survival and Overall Survival

ETS was associated with improved PFS [no ETS: 8.2 (7.6–8.8) months; ETS: 11.9 (10.2–13.5) months; log-rank *p* < 0.001] and OS [no ETS: 21.2 (18.8–23.6) months; ETS: 28.5 (25.2–31.8) months; log-rank *p* = 0.002]. These survival differences remained statistically significant when adjusted for treatment arm, sex, age, *RAS* mutation, and *BRAF* mutation [PFS: *p* < 0.001; HR 0.618 (95% CI 0.499–0.767); OS: *p* = 0.003; HR 0.713 (95% CI 0.568–0.895)].

**Figure 3:**
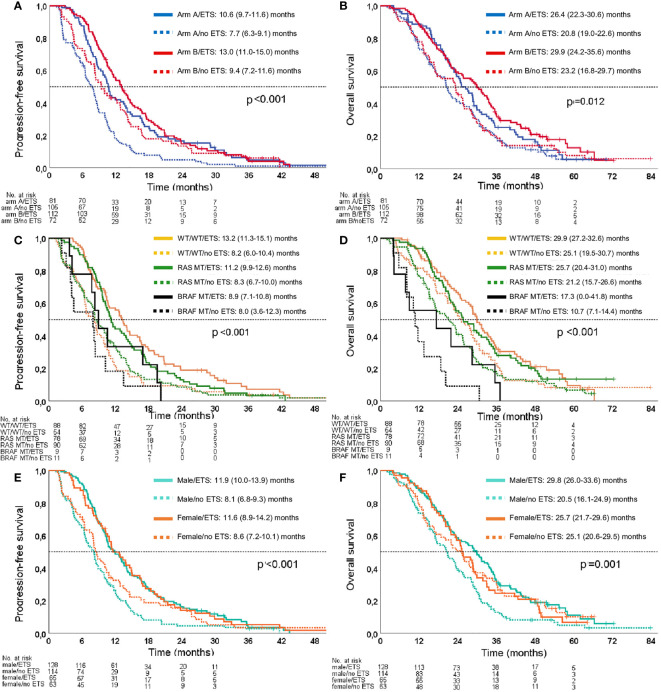
Kaplan–Meier estimates for the association of ETS with PFS and OS. **(A)** Association of ETS with PFS in the study arms. **(B)** Association of ETS with OS in the study arms. **(C)** Association of ETS with PFS in molecular subgroups. **(D)** Association of ETS with OS in molecular subgroups. **(E)** Association of ETS with PFS according to sex. **(F)** Association of ETS with OS according to sex. Arm A: sequential treatment arm; Arm B: initial combination treatment arm; WT/WT: *RAS/BRAF* WT subgroup.

ETS correlated with prolonged survival irrespective of treatment arm (PFS: log-rank *p* < 0.001; OS: log-rank *p* = 0.012) and molecular subgroup (PFS: log-rank *p* < 0.001; OS: log-rank *p* < 0.001). Please refer to **Figure 3** for the respective Kaplan–Meier curves.

ETS was significantly associated with survival benefit in male patients with regard to PFS [log rank *p* < 0.001; HR 0.532 (95% CI 0.409–0.692)] and OS [log rank *p* < 0.001; HR 0.574 (95% CI 0.437–0.756)]. However, this association could not be reproduced in female patients with regard to PFS [log rank *p* = 0.105; HR 0.745 (95% CI 0.521–1.066)] and OS [log rank *p* = 0.965; HR 1.009 (95% CI 0.685–1.486)] (**Figure 3**). These gender observations were also evident when the predictive effect of ETS was adjusted for treatment arm, age, *RAS* mutation, and *BRAF* mutation [PFS male: *p* < 0.001, HR 0.550 (95% CI 0.418–0.725); PFS female: *p* = 0.109, HR 0.734 (95% CI 0.503–1.072); OS male: *p* = 0.001, HR 0.617 (95% CI 0.465–0.891); OS female: *p* = 0.490, HR 0.868 (95% CI 0.581–1.297)].

## Discussion

The objective of this manuscript was to elucidate to which extent initial irinotecan in the context of fluoropyrimidines and bevacizumab improves early treatment response (ETS, DpR) as well as time to DpR as a novel endpoint related to these parameters. Additionally, subgroup analyses were performed to identify differences between molecular subtypes and sex.

In our analysis, ETS and DpR outcomes were more favorable in mCRC patients receiving initial combination treatment. The gain in ETS frequency and median DpR through the upfront use of irinotecan was 17.4% and 15.3%, respectively. These improvements are well comparable to the gains in ETS and DpR that are reported for other cytotoxic drugs, namely, anti-EGFR antibodies, in *RAS* WT mCRC (3, 11), suggesting that the potential of irinotecan to improve early outcome parameters and therefore also parameters depending on early responses, like secondary resectability of metastases, might be very similar to that of anti-EGFR antibodies.

The benefit in early response parameters was pronounced in the subpopulation of patients with *RAS*/*BRAF* WT tumors, indicating a particularly high sensitivity to irinotecan-containing treatment in these tumors. This finding may suggest that *RAS/BRAF* WT mCRC represents—unlike *RAS* MT mCRC—a generally treatment-sensitive subtype of mCRC that likely benefits from intensification of therapy.

Aside from molecular subgroups, sex also appears to impact early response parameters with male patients deriving a more substantial benefit from upfront irinotecan-containing therapy compared to female patients. It can be assumed that women as compared to men are either less sensitive to irinotecan or more sensitive to 5-FU/capecitabine. Since the expression of dihydropyrimidine dehydrogenase (DPD) is known to be lower in female colorectal cancer patients (19), it appears more likely that women might be more sensitive to 5-FU and its prodrug capecitabine (19). As DPD represents the rate-limiting enzyme in the catabolism of 5-FU, lower DPD expression levels lead to increased serum levels of 5-FU in female patients and might increase not only toxicity but also efficacy (20). Thus, the benefit of adding irinotecan to upfront chemotherapeutic treatment might not be as substantial as in the male population. This assumption remains a matter of debate and should therefore be a subject of future investigations given the fact that other potential effects of gender on clinical, histopathological, and therapeutic factors in colorectal cancer have not been considered in this analysis.

However, our observation on the less pronounced response of female patients to the upfront use of an intensified chemotherapeutic regimen is contrasted by the results of a retrospective analysis of the phase 3 trials TRIBE and TRIBE-2 demonstrating no sex differences considering the benefit from intensified chemotherapy in mCRC patients (21). Factors that may explain this discrepancy include the older population in XELAVIRI, a bias caused by the slightly different proportion of molecular subgroups (more *RAS* and *BRAF* MT patients in TRIBE and TRIBE-2), and the smaller number of patients in our analysis (21).

The time to DpR was significantly shorter in patients treated within the sequential therapy arm as compared to patients in the upfront combination treatment arm (4.4 vs. 5.1 months; *p* = 0.03), but with less DpR. In general, time to DpR does not seem to be a specifically sensitive endpoint in the context of the specific trial regimens and the molecular subgroups of mCRC. However, it might be noted that the time to DpR appears longer in the XELAVIRI trial as compared to other recent trials, maybe reflecting the limited frequency of trial dropouts due to secondary resectability of tumors (3, 22).

In the XELAVIRI trial, time to failure of strategy (TFS) was the primary study endpoint. TFS represents an alternative surrogate endpoint for the conventional survival endpoints PFS and OS and was shown to strongly correlate with these secondary study endpoints. Therefore, we analyzed the association of ETS with PFS and OS instead of TFS. In our analysis, ETS was predictive of PFS and OS regardless of treatment arm. Thus, ETS seems to be a suitable early response-associated prognostic marker for the initial use of fluoropyrimidine and bevacizumab combinations (with or without irinotecan) in mCRC patients, which is in accordance with the findings on anti-EGFR antibodies (11, 15–17), and other chemotherapeutic regimens (10, 12). Of note, whereas ETS was clearly associated with improved PFS and OS in male patients, ETS in female patients did not translate into a relevant survival benefit, potentially suggesting that ETS does not play an equally important role for the long-term outcome of female as compared to male mCRC patients in this study cohort comprising rather older patients.

The presented results are limited due to the retrospective nature of the analysis. In addition, there was a limited number of patients in the analyzed molecular subgroups and the generated hypotheses should be further evaluated in larger patient cohorts.

In conclusion, Irinotecan-based combination therapy as compared to sequential therapy with bevacizumab, respectively, improves early response parameters. Improvement in ETS and DpR appears pronounced in patients with *RAS*/*BRAF* WT mCRC and male patients, suggesting a high sensitivity to irinotecan-based treatment. In the XELAVIRI trial, ETS was associated with improved PFS and OS regardless of treatment arm. In accordance with the current literature, ETS seems to be a suitable prognostic marker for the initial use of fluoropyrimidine- and bevacizumab-based combinations in mCRC patients. However, in our cohort, this finding was rather driven by male than female patients, potentially indicating that ETS might be less predictive of long-term outcome in a female population of older patients.

## Data Availability Statement

The data analyzed in this study are subject to the following licenses/restrictions: With respect to the clinical trial “Sequential Versus Combination Therapy of Metastatic Colorectal Cancer Using Fluoropyrimidines, Irinotecan, and Bevacizumab: A Randomized, Controlled Study-XELAVIRI”, sponsor code AIO-KRK-0110, NCT01249638, the Ludwig-Maximilians-Universität (LMU) Munich (Germany) acting as the legal sponsor is committed to provide information about its results to researchers with the goal of facilitating scientific progress. Information that will be considered for disclosure includes individual participant data that underlie the results reported in this article (text, tables, figures, and appendices). Additionally, study protocol and statistical analysis plan can be made available. All data shared must be anonymized to protect the privacy of the patients who participated in the trial, in accordance with applicable laws and regulations and in compliance with the International Council for Harmonization and Good Clinical Practice (ICH/GCP). Researchers should provide a scientifically sound proposal for approval to gain access to the requested data. Shared data are only to be used to achieve aims of the approved proposal. Requests to access these datasets should be directed to volker.heinemann@med.uni-muenchen.de.

## Ethics Statement

The XELAVIRI study was performed in accordance with the Declaration of Helsinki. The protocol was approved by the ethics committees of all participating trial centers. All patients provided written informed consent before trial entry. A contract research organization (ClinAssess GmbH, Leverkusen, Germany) was responsible for randomization, data management, monitoring, and primary data analysis. Please refer to the primary publication for detailed information on ethics approval, the responsible ethics committee and consent from the participants (PMID: 30388045 DOI: 10.1200/JCO.18.00052). The patients/participants provided their written informed consent to participate in this study.

## Author Contributions

AK: Data analysis and interpretation, statistical analysis, manuscript preparation, manuscript editing, and manuscript review. VH: Study concepts, study design, data acquisition, data analysis and interpretation, manuscript editing, and manuscript review. LF: Data acquisition, manuscript editing, and manuscript review. TD: Data acquisition, manuscript editing, and manuscript review. FK: Data acquisition, manuscript editing, and manuscript review. JU: Data acquisition, manuscript editing, and manuscript review. MS: Data acquisition, manuscript editing, and manuscript review. JF-R: Data acquisition, manuscript editing, and manuscript review. BP: Data acquisition, manuscript editing, and manuscript review. CD: Data acquisition, manuscript editing, and manuscript review. UG: Data acquisition, manuscript editing, and manuscript review. KH: Data acquisition, manuscript editing, and manuscript review. SH: Data analysis, statistical analysis, manuscript editing, and manuscript review. AS: Data acquisition, manuscript editing, and manuscript review. AA: Data acquisition, manuscript editing, and manuscript review. IJ: Data acquisition, manuscript editing, and manuscript review. JE: Data acquisition, manuscript editing, and manuscript review. SS: Study concepts, study design, data acquisition, data analysis and interpretation, manuscript editing, and manuscript review. DPM: Study concepts, study design, data acquisition, data analysis and interpretation, manuscript preparation, manuscript editing, and manuscript review. All authors contributed to the article and approved the submitted version.

## Funding

The XELAVIRI trial was legally sponsored by the Ludwig-Maximilians-University (LMU) Munich (Germany) with financial support of Roche Pharma AG. The financial supporter had no role in the design, data collection, analysis, interpretation of the data, writing or decision to submit the manuscript for publication.

## Conflict of Interest

AK: Honoraria: Taiho Pharmaceutical, Servier; Travel, Accommodations, Expenses: Roche, Medac. VH: Honoraria: Roche, Celgene, Amgen, Sanofi, Merck, Sirtex Medical, Baxalta, Eli Lilly, Boehringer Ingelheim, Taiho Pharmaceutical, Servier; Consulting or Advisory Role: Merck, Amgen, Roche, Sanofi, Boehringer Ingelheim, Celgene, Sirtex Medical, Baxalta, Servier, Halozyme, MSD, Bristol-Myers Squibb; Research Funding: Merck (Inst), Amgen (Inst), Roche (Inst), Celgene (Inst), Boehringer Ingelheim (Inst), Sirtex Medical (Inst), Shire (Inst); Travel, Accommodations, Expenses: Merck, Roche, Sirtex Medical, Amgen, Servier, Shire, MSD, Bristol-Myers Squibb. LF: Honoraria: Novartis, Roche, Sanofi; Travel, Accommodations, Expenses: Amgen. TD: Consulting or Advisory Role: Novartis. CD: Honoraria: Janssen, Novartis, Celgene, Incyte; Consulting or Advisory Role: Abbvie, Bayer; Travel, Accommodations, Expenses: Merck. UG: Honoraria: Servier, Boehringer Ingelheim, Sirtex Medical, Daiichi Sankyo; Consulting or Advisory Role: Novartis, Merck, Amgen, Hexal, Bristol-Myers Squibb;Travel, Accommodations, Expenses: Merck, Amgen. AS: Honoraria: Roche, Servier/Taiho; Travel, Accommodations, Expenses: Roche, Merck KGaA, MSD Sharp & Dohme, Pfizer, Amgen. KH: Honoraria: Roche; Travel, Accommodations, Expenses: AMGEN, Celgene, Lilly. SH: Employed: ClinAssess GmbH. AA: Consulting or Advisory Role: Roche; Travel, Accommodations, Expenses: Pfizer, Roche, Eli Lilly, Novartis, PharmaMar. JE: Honoraria: Merck, Roche, Amgen, Sanofi, Pierre-Fabre, Servier, Taiho, BMS, Eisai, Novartis; Consulting or Advisory Role: Amgen, Pierre-Fabre, BMS, Servier; Travel, Accommodations, Expenses: AstraZeneca, Apceth. SS: Honoraria: AMGEN, Bayer, BMS, ESAI, Lilly, Merck KGaA, MSD, Pierre-Fabre, Roche, Sanofi, Servier, Taiho, Takeda; Consulting or Advisory Role: AMGEN, Bayer, BMS, ESAI, Lilly, Merck KGaA, MSD, Pierre-Fabre, Roche, Sanofi, Servier, Taiho, Takeda; Travel, Accommodations, Expenses: Merck, Roche, Sanofi, Bayer, Sirtex Medical, Amgen, Eli Lilly, Takeda, Pierre Fabre. CG-J: Travel, Accommodations, Expenses: Roche. DPM: Honoraria: Merck Serono, Amgen, Roche, Servier, Bristol-Myers Squibb, Pfizer, Sirtex Medical; Consulting or Advisory Role: Merck Serono, Amgen, Bayer; Research Funding: Merck Serono (Inst), Roche (Inst), Amgen (Inst); Travel, Accommodations, Expenses: Amgen, Merck Serono, Bayer, Servier, Bristol-Myers Squibb.

The remaining authors declare that the research was conducted in the absence of any commercial or financial relationships that could be construed as a potential conflict of interest.

## Publisher’s Note

All claims expressed in this article are solely those of the authors and do not necessarily represent those of their affiliated organizations, or those of the publisher, the editors and the reviewers. Any product that may be evaluated in this article, or claim that may be made by its manufacturer, is not guaranteed or endorsed by the publisher.

## Abbreviations

Bev, Bevacizumab; BRAF, v-Raf murine sarcoma viral oncogene homolog B; DPD, Dihydropyrimidine dehydrogenase; DpR, Depth of response; EGFR, Epidermal growth factor receptor; ETS, Early tumor shrinkage; FP, Fluoropyrimidine; HR, Hazard ratio; Iri, Irinotecan; mCRC, Metastatic colorectal cancer; mITT, Modified intention-to-treat; MT, Mutant; ORR, Objective response rate; OS, Overall survival; PFS, Progression-free survival; RAS, Rat sarcoma; TFS, Time to failure of strategy; WT, Wild type.
