# Supplementary material for: Response and Disease Dynamics in Untreated Metastatic Colorectal Cancer With Bevacizumab-Based Sequential vs. Combination Chemotherapy—Analysis of the Phase 3 XELAVIRI Trial
Source: Front Oncol. 2022 Feb 18;12:751453. doi: 10.3389/fonc.2022.751453 (PMC8895369; doi:10.3389/fonc.2022.751453)
Supplement: Supplementary file 1 [file DataSheet_1.pdf]

**Supplementary TABLE A.1: Treatments used in the XELAVIRI/ AIO KRK0110 trial.**

<table>
<tr><th rowspan="3">Sequential therapy arm</th><th>Induction</th><th>Escalation</th></tr>
<tr><td>oral capecitabine 1250 mg/m$^2$ twice daily, days 1-14 plus infusional bevacizumab 7.5 mg per kg of body weight on day 1, repeated every 3 weeks</td><td>capecitabine 800 mg/m$^2$ twice daily days 1-14, intravenous irinotecan 200 mg/m$^2$ on day 1 plus bevacizumab at a dose of 7.5 mg per kg body weight infused on day 1, repeated every 3 weeks</td></tr>
<tr><td>intravenous on day 1: racemic folinic acid with 400 mg/m$^2$, 5-FU bolus of 400 mg/m$^2$, fluorouracil over 46 hours of 2400 mg/m$^2$ bevacizumab 5 mg per kg body weight; repeated every 2 weeks</td><td>intravenous on day 1: irinotecan 180 mg/m$^2$, racemic folinic acid with 400 mg/m$^2$, 5-FU bolus of 400 mg/m$^2$, fluorouracil over 46 hours of 2400 mg/m$^2$ bevacizumab 5 mg per kg body weight; repeated every 2 weeks</td></tr>
<tr><th rowspan="3">Combination therapy arm</th><th>Induction</th><th>Intermittent de-escalation<br>(in case of at least stable disease for more than six months)</th></tr>
<tr><td>capecitabine 800 mg/m$^2$ twice daily days 1-14, intravenous irinotecan 200 mg/m$^2$ on day 1 plus bevacizumab at a dose of 7.5 mg per kg body weight infused on day 1, repeated every 3 weeks</td><td>oral capecitabine 1250 mg/m$^2$ twice daily, days 1-14 plus infusional bevacizumab 7.5 mg per kg of body weight on day 1, repeated every 3 weeks</td></tr>
<tr><td>intravenous on day 1: irinotecan 180 mg/m$^2$, racemic folinic acid with 400 mg/m$^2$, 5-FU bolus of 400 mg/m$^2$, fluorouracil over 46 hours of 2400 mg/m$^2$ bevacizumab 5 mg per kg body weight; repeated every 2 weeks</td><td>intravenous on day 1: racemic folinic acid with 400 mg/m$^2$, 5-FU bolus of 400 mg/m$^2$, fluorouracil over 46 hours of 2400 mg/m$^2$ bevacizumab 5 mg per kg body weight; repeated every 2 weeks</td></tr>
</table>

**Supplementary FIGURE A.1: Consort diagram of study population.**

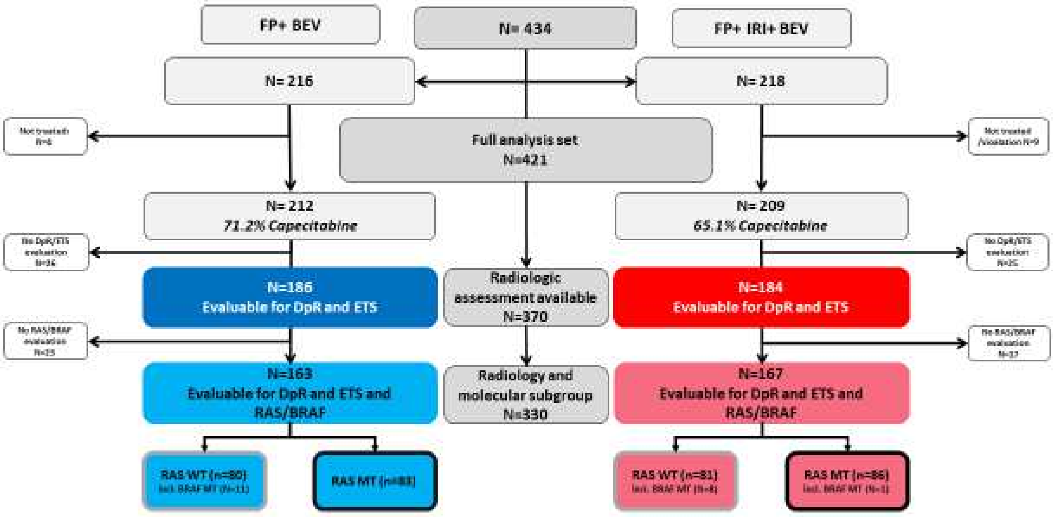


**Legend:** FP- fluoropyrimidine; BEV- bevacizumab; IRI- irinotecan; WT- wildtype; MT- mutant.

**Supplementary FIGURE A.2: Best response in the trial.**

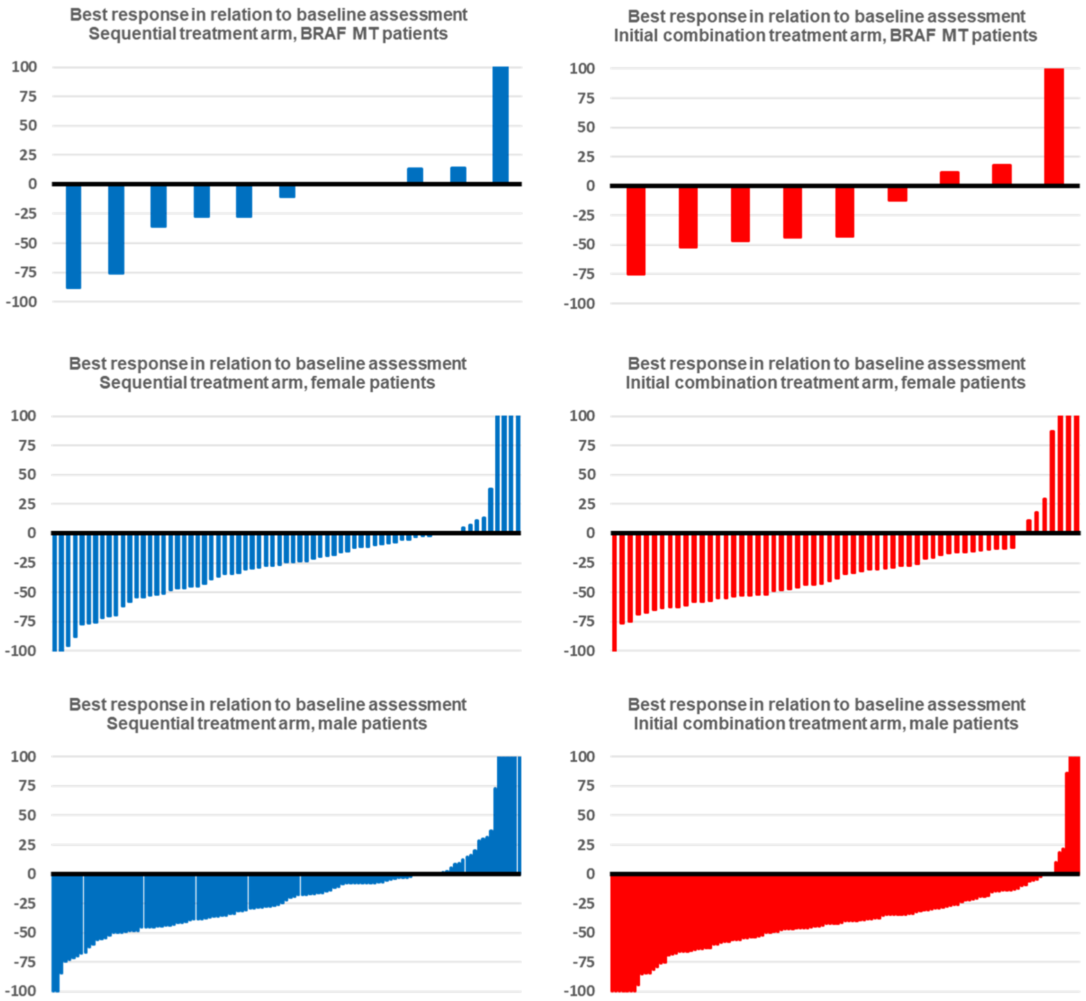


**Legend:** Blue images display response assessments of the sequential treatment arm (fluoropyrimidine plus bevacizumab), red images show response assessments of the initial combination treatment arm (fluoropyrimidine, bevacizumab, and irinotecan) in (from top to bottom) groups: BRAF mutant, female patients, male patients.
